# Alpha-Synuclein and Microglia in Parkinson’s Disease: From Pathogenesis to Therapeutic Prospects

**DOI:** 10.3390/jcm13237243

**Published:** 2024-11-28

**Authors:** Hyemi Eo, Sehwan Kim, Un Ju Jung, Sang Ryong Kim

**Affiliations:** 1 School of Life Science and Biotechnology, BK21 FOUR KNU Creative BioResearch Group, Kyungpook National University, Daegu 41566, Republic of Korea; ahm2084@knu.ac.kr (H.E.); arputa@naver.com (S.K.); 2 Department of Food Science and Nutrition, Pukyong National University, Busan 48513, Republic of Korea; jungunju@pknu.ac.kr; 3 Brain Science and Engineering Institute, Kyungpook National University, Daegu 41404, Republic of Korea

**Keywords:** Parkinson’s disease, alpha-synuclein, microglia, neuroinflammation, propagation

## Abstract

Parkinson’s disease (PD) is a neurodegenerative disorder characterized by both motor symptoms and non-motor features. A hallmark of PD is the misfolding and accumulation of alpha-synuclein (α-syn), which triggers neuroinflammation and drives neurodegeneration. Microglia, brain cells that play a central role in neuroinflammatory responses and help clear various unnecessary molecules within the brain, thus maintaining the brain’s internal environment, respond to α-syn through mechanisms involving inflammation, propagation, and clearance. This review delves into the complex interplay between α-syn and microglia, elucidating how these interactions drive PD pathogenesis. Furthermore, we discuss emerging therapeutic strategies targeting the α-syn–microglia axis, with a focus on modulating microglial functions to mitigate neuroinflammation, enhance clearance, and prevent α-syn propagation, emphasizing their potential to slow PD progression.

## 1. Introduction

Parkinson’s disease (PD) is the second most prevalent neurodegenerative disorder, with rising incidence due to global population aging, presenting an increasing burden on healthcare systems worldwide [1,2]. Patients with PD exhibit motor symptoms such as bradykinesia, rigidity, and tremors, along with non-motor symptoms, including constipation, hyposmia, depression, cognitive decline, and sleep alternation [3]. The pathophysiology of PD is influenced by an interplay between several mechanisms, including abnormal α-synuclein (α-syn) aggregation, mitochondrial dysfunction, lysosomal or vesicle transport deficiencies, synaptic transport issues, and neuroinflammation [3,4]. These processes contribute to the accelerated degeneration of the nigrostriatal dopaminergic pathway and impact other motor and non-motor circuits, leading to the diverse symptoms observed in patients with PD [3,4]. A central pathological process of PD is the accumulation of α-syn, which plays a crucial role in synaptic vesicle regulation and neurotransmitter release under normal conditions [5,6,7,8,9]. However, in PD, α-syn misfolds and aggregates into Lewy bodies contributing to neuroinflammation, further exacerbating neuronal damage and disease progression [10,11,12,13].

Microglia, the resident immune cells in the brain, play a role in the progression of PD. Microglia become activated in response to α-syn aggregates, leading to chronic neuroinflammation that exacerbates neurodegeneration [14]. Microglia also mediate the clearance of these aggregates through phagocytosis and autophagy; however, these clearance systems are often impaired in PD [15,16]. Moreover, microglia can propagate α-syn aggregates facilitating the spread of pathology throughout the brain [17,18,19,20]. Therefore, understanding this balance between activation, propagation, and clearance roles is crucial for developing therapeutic strategies that target microglial function in PD.

Recent advancements in therapeutic research targeting the interplay between microglia and α-syn have been conducted at both the preclinical and clinical levels [21]. Strategies such as inhibiting α-syn propagation, modulating microglial activity, and leveraging personalized gene therapies can potentially mitigate neuroinflammation and slow neurodegeneration [22,23,24]. Furthermore, diagnostic tools, such as PET imaging and AI-based models, can facilitate the monitoring of disease progression and therapeutic efficacy, as well as our understanding and management of PD [25,26,27,28]. These approaches represent a promising shift toward more precise, targeted interventions in PD treatment.

This review explores the intricate interplay between α-syn and microglia in PD, examining their roles in disease pathogenesis and discussing potential therapeutic strategies to modulate these interactions to slow or halt disease progression.

## 2. Role of α-Syn in PD

The physiological functions and structural flexibility of α-syn are essential for maintaining synaptic and mitochondrial homeostasis. Meanwhile, pathological modifications and aggregation of α-syn drive its transition into toxic forms, promoting neurodegeneration and contributing to PD progression. This session highlights the importance of the structural dynamics and modifications of α-syn in disease progression.

### 2.1. α-Syn: Structure and Physiological Function

α-Syn is a small, 140-amino-acid protein predominantly expressed in the central nervous system, particularly in presynaptic nerve terminals [6]. In physiological conditions, α-syn primarily exists in an unstructured or intrinsically disordered state but can adopt different conformations, including α-helices upon membrane binding or β-sheets during aggregation [29]. Functionally, α-syn is involved in regulating synaptic vesicle trafficking and neurotransmitter release. In these processes, α-syn binds to synaptic vesicles and promotes their clustering, contributing to the maintenance of a reserve pool of synaptic vesicles [5,6]. Moreover, α-syn interacts with the SNARE complex, essential for synaptic vesicle docking and fusion, contributing to neurotransmitter release and synaptic plasticity [7,8]. Additionally, α-syn is implicated in membrane curvature sensing and stabilization, essential for synaptic vesicle recycling, and plays a role in maintaining mitochondrial function and bioenergetics, which is crucial for neuronal survival [9].

Structurally, α-syn has three key regions: the N-terminal region, non-amyloid-β component (NAC) domain, and C-terminal domain. The N-terminal region comprises 11-residue repeats with a conserved KTKEGV motif, which facilitates the formation of an amphipathic α-helix when bound to lipid membranes [29]. The central NAC domain is crucial for the aggregation properties of α-syn, as it promotes the formation of β-sheet structures, a critical step in the aggregation process that leads to the formation of toxic oligomers and fibrils [30,31,32]. The C-terminal domain is highly acidic, proline-rich, and largely disordered. Furthermore, the C-terminal domain interacts with other proteins and metal ions, and its flexibility is thought to help maintain α-syn in a soluble, non-toxic state [29,33]. Under physiological conditions, α-syn primarily exists in an intrinsically disordered state but can adopt an amphipathic α-helix conformation upon membrane binding. However, conformational changes occur in pathological conditions, particularly in the NAC domain, driving toxic oligomer conformation [29,32]. These β-sheet-rich oligomers stack together to form the core of fibrils, which are highly insoluble and toxic. The process begins with α-syn monomers adopting an extended conformation that facilitates NAC–NAC interactions, forming β-sheets [29,32]. When α-syn interacts with lipid membranes, the N-terminal region stabilizes the α-helical conformation. However, when these interactions are disrupted, α-syn may shift toward β-sheet formation, enhancing aggregation [34]. While flexible and disordered under normal conditions, the C-terminal region can also influence aggregation when modified by post-translational modifications (PTMs), such as phosphorylation at serine 129 (Ser129), which accelerates fibril formation and promotes neurotoxicity [35]. During the aggregation process, α-syn undergoes intermediate stages, including the formation of soluble oligomers that are believed to be particularly neurotoxic, before forming insoluble fibrils.

### 2.2. Modification of α-Syn in PD: Triggers of Aggregation and Disease Progression

The pathogenic role of α-syn in PD largely depends on its aggregation state. α-Syn aggregates are a major component of Lewy bodies, the pathological inclusions observed in PD. Lewy bodies disrupt cellular functions by sequestering vital proteins and impairing cellular waste disposal mechanisms, contributing to neurodegeneration [36]. In contrast to monomeric α-syn, which is non-toxic and soluble, the aggregation of α-syn represents a toxic gain of function, often associated with mutations in the *SNCA* gene, which encodes α-syn [10]. The connection between α-syn and PD was established in 1997 when point mutations in the *SNCA* gene were identified in familial PD cases (PARK1 locus) [37]. Six missense mutations in SNCA, i.e., Ala53Thr (A53T), Ala30Pro (A30P), Glu46Lys (E46K), His50Gln (H50Q), Gly51Asp (G51D), and Ala53Glu (A53E), have been linked to autosomal dominant forms of PD, each contributing to different aspects of aggregation and early onset symptoms [37,38,39,40,41,42,43]. A53T and A53E lead to early-onset PD by enhancing α-syn fibril formation and causing rapid disease progression with severe motor symptoms [37,43]. A30P disrupts membrane binding, reduces the stability of the α-helical structure, and accelerates oligomerization, contributing to PD pathology [39]. E46K promotes α-syn aggregation and fibril formation, leading to early-onset dementia with Lewy bodies (DLB) [40]. H50Q also increases protein aggregation and has been linked to both PD and DLB [41,42]. Lastly, G51D has been linked to atypical PD with pyramidal tract symptoms and slower aggregation, leading to a unique clinical presentation [38].

Furthermore, PTMs of α-syn, such as phosphorylation, nitration, acetylation, and ubiquitination, play a crucial role in modulating the pathological states and aggregation potential of α-syn [44]. Among the PTMs, phosphorylation at Ser129 is the most well-known associated with α-syn aggregation and is commonly found in Lewy bodies [35,45,46]. While Ser129 phosphorylation promotes fibril formation and neurotoxicity, phosphorylation at Ser87 may have protective effects by reducing aggregation propensity [45]. Other key PTMs include nitration, acetylation, and ubiquitination. Indeed, nitration, particularly at tyrosine residues, promotes oligomer formation and disrupts normal folding, exacerbating oxidative stress and neurotoxicity [47]. Nitrated α-syn is found in Lewy bodies and can enhance the pathological progression of PD by accelerating the misfolding and aggregation of α-syn [48,49]. Conversely, acetylation at the N-terminal region stabilizes α-syn and can potentially reduce its aggregation. However, aberrant acetylation may still alter the normal function of α-syn and contribute to its pathological misfolding [50,51]. Ubiquitination of α-syn is a critical PTM that typically marks the protein for degradation via the ubiquitin–proteasome system (UPS). However, in PD, selective dysfunction of UPS results in the accumulation of misfolded or aggregated α-syn. Ubiquitinated α-syn is frequently observed in Lewy bodies, the pathological inclusions characteristic of PD [52]. Disruptions in the ubiquitination process contribute to α-syn aggregation and neurotoxicity, thereby accelerating the progression of PD [52]. Collectively, these modifications regulate the structural dynamics of α-syn and its transition from a soluble, non-toxic form to an aggregated, pathogenic state, thus making them critical factors in PD progression.

## 3. Interplay Between α-Syn and Microglia in PD

**Figure 1:**
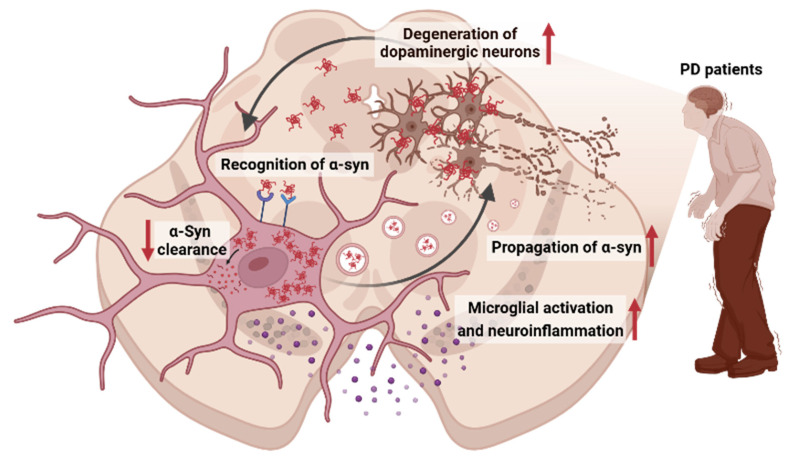
A schematic that illustrates the key interaction mechanisms between microglia and α-syn in PD: Bidirectional interaction between α-syn and microglia. Microglia recognize α-syn aggregates through surface receptors, leading to their internalization. Once inside microglia, impaired clearance mechanisms may result in the accumulation of α-syn, which can then be released, facilitating the propagation of pathological aggregates to neighboring cells. The internalized α-syn triggers microglial activation, resulting in the production and release of pro-inflammatory cytokines, contributing to neuroinflammation. The persistent inflammatory response, alongside the spread of α-syn aggregates, ultimately leads to the degeneration of dopaminergic neurons, a hallmark of PD progression. (Created and modified using BioRender.com license no: RO27IGFJC2).

Microglia, the resident immune cells in the central nervous system (CNS), originate from the embryonic yolk sac. In their resting state, they continuously monitor the brain environment, responding to infections, injuries, and cellular debris. Furthermore, microglia play a crucial role in maintaining brain homeostasis through various processes such as synaptic pruning [53], neurogenesis [54], and immune surveillance [55]. Beyond these homeostatic roles, microglia can also recognize and respond to α-syn aggregates, a hallmark of PD. The bidirectional relationship between α-syn and microglia underlies the complex inflammatory responses seen in PD; α-syn aggregates activate microglia, and in turn, activated microglia can either promote α-syn clearance or exacerbate neuroinflammation [16] (Figure 1). This complex interplay is crucial in understanding the progression of PD and developing potential therapeutic strategies targeting the modulation of microglial function.

### 3.1. Recognition of α-Syn Aggregates by Microglia

Microglia recognize and bind extracellular α-syn aggregates through diverse pattern recognition receptors (PRRs), which are pivotal for initiating the microglial response.

Toll-like receptors (TLRs), a family of PRRs, are crucial components in the innate immune system that detect pathogen-associated molecular patterns and damage-associated molecular patterns [56]. TLR2 and TLR4 detect α-syn aggregates, particularly oligomers. TLR2 shows a higher sensitivity to these soluble forms, which are abundant in the early stages of PD, while TLR4 can recognize multiple forms of α-syn, including fibrils [57]. Upon activation, both TLR2 and TLR4 initiate the nuclear factor-κB (NF-κB) pathway, leading to the release of pro-inflammatory cytokines and contributing to neuroinflammation [57,58,59]. Interestingly, TLR4 has also been implicated in this process of autophagy, which aids in the clearance of α-syn aggregates. This suggests that TLR4 activation not only promotes inflammation but also facilitates the degradation of toxic α-syn species, highlighting its dual role in PD pathology [60].

The Fcγ receptors (FcγRs) are also critical regulators of immune responses in microglia, particularly in recognizing and responding to immunoglobulin G (IgG)-opsonized complexes such as α-syn aggregates. These receptors modulate microglial activation and balance phagocytosis and inflammation [13,61,62]. FcγRI, a high-affinity receptor, binds to IgG-opsonized α-syn aggregates and triggers the phosphorylation of immunoreceptor tyrosine-based activation motifs activating downstream inflammatory signaling pathways [13,61]. By contrast, FcγRIIB, a low-affinity receptor, downregulates immune responses and suppresses phagocytosis. FcγRIIB contains immunoreceptor tyrosine-based inhibitory motifs (ITIMs) in its cytoplasmic domain. Upon α-syn binding, the ITIMs are phosphorylated, interfering with intracellular signaling cascades necessary for actin polymerization and the engulfment of α-syn aggregates [13,62]. This balanced interaction between FcγRI and FcγRIIB is critical in modulating microglial responses to α-syn.

Scavenger receptors recognize oxidized or aggregated forms of α-syn that can induce microglial activation, phagocytosis, and reactive oxygen species (ROS) production. Cluster of differentiation 36 (CD36), a class B scavenger receptor expressed on the surface of microglia, plays a significant role in recognizing and binding α-syn aggregates [63,64]. CD36 specifically recognizes oxidized forms of α-syn, commonly found in the brains of patients with PD. Upon binding to α-syn, CD36 promotes internalization of α-syn and mediates activation of downstream inflammatory signaling pathways, leading to the production of ROS [65]. Other scavenger receptors, such as scavenger receptor class A1 and macrophage antigen complex-1 (Mac-1), have been reported to mediate α-syn-induced microglial activation [64,66]. Zhang et al. demonstrated that α-syn directly interacts with Mac-1 receptors, which subsequently activate NADPH oxidase, resulting in increased ROS production and promoting neuroinflammation [66].

Additionally, the receptors for advanced glycosylation end products (RAGE), a PRR expressed on microglia, can recognize and bind various ligands, including α-syn aggregates [67]. In the context of PD, RAGE specifically binds to misfolded and aggregated forms of α-syn, particularly fibrillary α-syn, which are abundant in the diseased state. Upon binging to α-syn fibrils, RAGE triggers the initiation and activation of the NF-κB signaling pathway [68,69]. Activation of RAGE also stimulates the production of pro-inflammatory cytokines and chemokines, which further recruit immune cells to the site of α-syn accumulation, amplifying neuroinflammatory responses. Moreover, the interaction between RAGE and α-syn fibrils may contribute to the chronic nature of neuroinflammation in PD, as RAGE signaling is sustained in response to ongoing α-syn aggregation [67].

Triggering receptor expressed on myeloid cells 2 (TREM2), a receptor expressed on microglia, plays a key role in regulating microglial responses, including phagocytosis and inflammation. Yin et al. demonstrated that microglia respond to the presence of α-syn aggregates via TREM2, potentially modulating their activity to prevent chronic neuroinflammation [70]. This response contributes to an adaptive microglial activation state that helps to maintain homeostasis and counteract neurotoxic effects in PD [70]. Other studies have shown that TREM2 deficiency in microglia exacerbates α-syn accumulation, intensifying neuroinflammation and worsening neurodegeneration [71,72].

### 3.2. Propagation of α-Syn Aggregates

**Figure 2:**
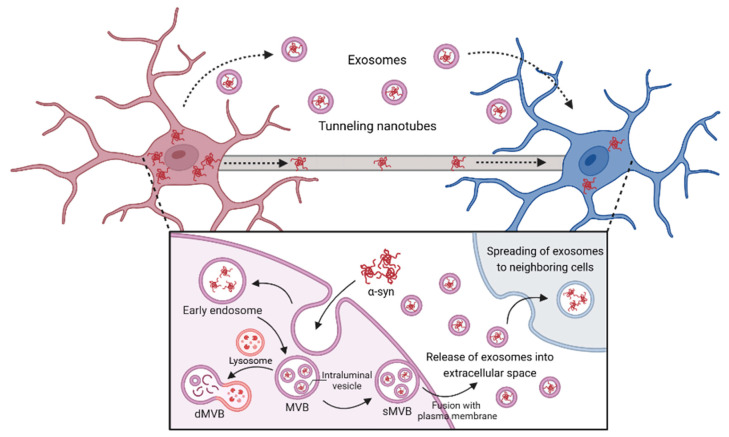
α-Syn propagation to neighboring cells by TNTs and exosomes of microglia. Microglia internalize extracellular α-syn via endocytosis and form early endosomes. The endosomal membrane then invaginates into its lumen and forms multivesicular bodies (MVBs). These mature MVBs are processed by two pathways: the degrative and secretory pathway. Degrative MVBs (dMVBs) fuse with lysosome, leading to degrade α-syn within MVBs; however, secretory MVBs (sMVBs) are fused with plasma membrane and then released into the extracellular space. Additionally, TNTs directly connect adjacent cells, enabling the transfer of α-syn aggregates across cytoplasmic bridges. Together, exosome-mediated and TNT-driven transport enhance the dissemination of α-syn pathology along neural networks, contributing to disease progression. (Created and modified using BioRender.com license no: WW27IGFNZL).

Microglia not only internalize α-syn but actively participate in its propagation, contributing to the spread of pathology within the CNS through various mechanisms, such as microglial exosomes and tunneling nanotubes (TNTs) (Figure 2). Exosomes are extracellular nanovesicles that carry cargo, including mRNA, lipids, and proteins, which are taken up by neighboring cells [73]. Microglia package internalized α-syn into exosomes, facilitating intercellular communication. These exosomes are then released into the extracellular space and subsequently taken up by surrounding neurons, astrocytes, and other microglia, promoting the spread of the α-syn pathology [19,74]. Studies indicate that α-syn-containing exosomes play a critical role in the propagation of α-syn and contribute to neuroinflammation and neurodegeneration related to PD pathology [75,76,77]. Indeed, elevated levels of CNS-derived exosomal α-syn have been detected in the plasma of PD patients [75]. Chang et al. demonstrated that exosomes containing α-syn were significantly released following α-syn treatment in BV-2 cells. These exosomes were then taken up by dopaminergic neurons, leading to neuronal death, secretion of pro-inflammatory cytokines, and microglial activation [76]. Interestingly, microglial exosomes are heterogeneous, with certain exosomal markers, such as CD9, CD63, and CD81 specifically enriched in α-syn-laden exosomes. This selective targeting could help explain the specificity in α-syn propagation pathways and may offer a basis for future therapeutic interventions [78]. Additionally, lysosomal dysfunction induced intracellular accumulation of α-syn, promoting the secretion of overloaded α-syn via exosomes. This indicates that the lysosomal dysfunction observed in PD leads to increased exosomal secretion and propagation of α-syn, ultimately exacerbating PD pathology [77].

Microglia actively communicate with other cells via specialized structures known as TNTs, thin membrane channels composed of F-actin. TNTs facilitate the direct cell-to-cell transfer of α-syn fibrils between microglia and other cells [79]. It has been reported that interactions between microglia and other cells through TNTs can simultaneously promote the propagation of α-syn and facilitate its degradation by distributing α-syn among microglia for degradation [20,80,81,82]. Formation of microglial TNTs can be increased when stimulated by inflammatory stimuli, and activated microglia transfer α-syn to neighboring cells. This communication enables the propagation of α-syn aggregates, potentially intensifying the inflammatory response and promoting the spread of pathology throughout the brain [20,80]. Furthermore, spreading α-syn through TNTs leads to lysosomal dysfunction, promoting microglia–microglia interactions via the TNTs [81]. Conversely, Scheiblich et al. demonstrated that microglia–microglia interactions through the TNTs distribute α-syn fibrils, lowering the individual burden and enhancing the efficiency of α-syn degradation [82]. In summary, microglial TNTs play a dual role in propagating α-syn pathology across the CNS and distributing α-syn fibrils for efficient degradation, underscoring their complex but crucial involvement in the progression of PD.

### 3.3. Activation of Inflammatory Pathways by α-Syn Aggregates

**Figure 3:**
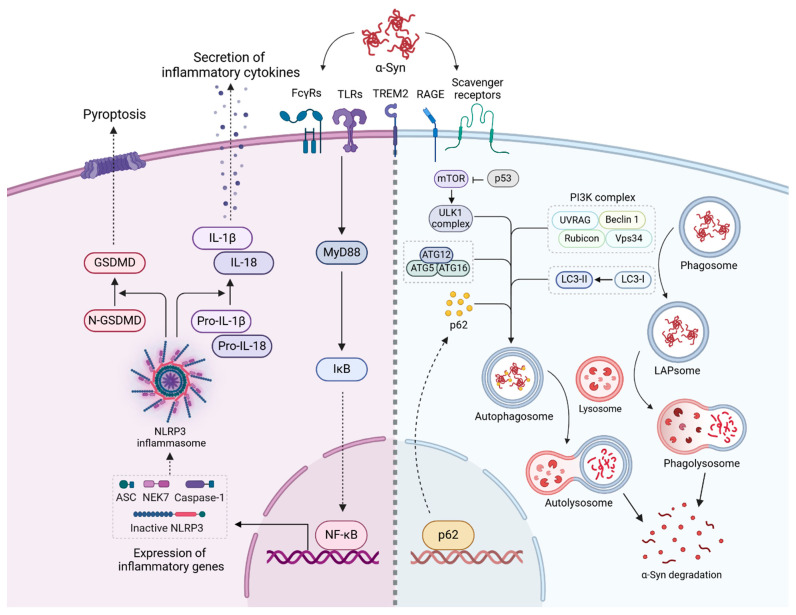
Mechanisms of microglial activation and α-syn degradation in PD. This schematic illustrates the intracellular signaling pathways through which microglia respond to α-syn aggregates, encompassing inflammatory activation, phagocytosis, and autophagy. Recognition of α-syn by microglial surface receptors activates MyD88, leading to the degradation of IκB and activation of NF-κB, which promotes inflammatory gene transcription. In parallel, the NLRP3 inflammasome becomes activated, assembling components such as ASC, NEK7, and caspase-1. Caspase-1 processes pro-IL-1β and pro-IL-18 into their mature, secreted forms (IL-1β and IL-18), further driving the inflammatory response. Activated GSDMD forms membrane pores, inducing pyroptotic cell death, a process implicated in chronic neuroinflammation and neurodegeneration in PD. Microglia degrade α-syn aggregates through phagocytosis and autophagy pathways. α-Syn internalized into phagosomes can undergo LAP, facilitated by the PI3K complex (UVRAG, Beclin-1, Rubicon, Vps34) to form phagolysosome α-syn degradation. Concurrently, the autophagy pathway, regulated by the ULK1 complex and modulated by mTOR and p53, encapsulates α-syn in autophagosomes. These autophagosomes fuse with lysosomes to form autolysosomes, where α-syn is degraded. The LC3 conjugation system (ATG5, ATG12, ATG16) and p62 serve as key components for cargo recognition and delivery. (Created and modified using BioRender.com license no: RN27JNRZ0N).

The interaction between microglia and α-syn aggregates triggers various inflammatory pathways, amplifying neuroinflammation in PD [83] (Figure 3). Upon recognizing α-syn aggregates, microglial receptors, such as TLRs, activate downstream signaling pathways that initiate the canonical IκB kinase complex through the myeloid differentiation primary response gene-88 (MyD88) adapter protein cascade [84]. This cascade leads to the phosphorylation and degradation of IκB, allowing for the nuclear translocation of NF-κB and the transcription of pro-inflammatory cytokine genes, including TNF-α, IL-1β, and IL-6 [84]. The NF-κB pathway is thus central to microglial activation and promotes sustained neuroinflammatory responses, contributing to the progression of PD pathology.

Activation of NF-κB also promotes upregulation of the NOD-like receptor protein 3 (NLRP3) inflammasome components. The NLRP3 inflammasome is an oligomeric complex that requires a secondary stimulus for activation [85,86]. Once activated, the NLRP3 complex catalyzes the conversion of inactive pro-caspase-1 and pro-IL-1β into their active forms, caspase-1 and pro-IL-1β, respectively [85,86]. The activated caspase-1 further cleaves the N-terminal fragment of gasdermin-D (GSDMD), a critical effector in pyroptosis. Cleaved GDSMD forms membrane pores, facilitating the release of active pro-inflammatory cytokines, such as IL-1β and IL-18, into the extracellular space and promoting an inflammatory cell death mechanism known as pyroptosis [87,88]. In particular, the binding of α-syn to TLRs and CD36 serves as a priming signal for NLRP3 activation, effectively linking α-syn recognition to inflammasome activity [15,65].

In addition, receptors such as CD36 and RAGE, upon α-syn binding, stimulate the production of ROS through NADPH oxidase activation. Elevated ROS levels create oxidative stress, which damages microglia and activates redox-sensitive pathways, including NF-κB, further enhancing cytokine production [16,65,69]. This interplay between ROS generation and NF-κB signaling establishes a positive feedback loop, exacerbating neuroinflammation and potentiating further oxidative damage to neurons. This ROS-driven amplification of NF-κB signaling contributes to a chronic inflammatory state, which is characteristic of neurodegenerative diseases like PD.

Chronic α-syn accumulation also induces ER stress within microglia, activating the unfolded protein response (UPR). Persistent UPR activation sensitizes microglia to an inflammatory state, potentially amplifying their response to α-syn aggregates and contributing to the inflammatory cycle in PD. Therefore, UPR activation in microglia can potentially intersect with these inflammatory pathways, as the IRE1 branch of the UPR can enhance NF-κB signaling, amplifying cytokine production and contributing to the chronic inflammation characteristic of PD [89,90].

In summary, the interaction between α-syn and microglial receptors initiates multiple inflammatory pathways, including NF-κB and NLRP3 inflammasome activation, which are amplified by ROS production. Furthermore, UPR activation induced by α-syn aggregates drives microglia into a chronic inflammatory state. Targeting these pathways offers potential therapeutic strategies to mitigate neuroinflammation and slow PD progression.

### 3.4. Clearance of α-Syn Aggregates

In PD, microglial mechanisms such as phagocytosis and autophagy are central to the clearance of α-syn aggregates. These pathways are crucial for maintaining cellular homeostasis and preventing the toxic buildup of misfolded proteins; however, their dysfunction in PD contributes to disease progression (Figure 3).

#### 3.4.1. Clearance of α-Syn Aggregates Through Phagocytosis and Autophagy in Microglia

Microglial phagocytosis is a process where microglia engulf extracellular α-syn aggregates. This process is initiated when PRRs, such as TLRs, FcγRs, and scavenger receptors, recognize α-syn aggregates on the surface of microglia [91]. These receptors activate intracellular signaling pathways, including the phosphoinositide 3-kinase (PI3K) pathway and small GTPases, such as Rac1, leading to actin cytoskeleton reorganization and phagosome formation [92]. Once phagosomes encapsulate α-syn aggregates, they fuse with lysosomes to form phagolysosomes, which are enriched with lysosomal enzymes that degrade α-syn into smaller components [93].

In addition to phagocytosis, microglia utilize the autophagy–lysosomal pathway to eliminate intracellular α-syn aggregates [15,94]. This process involves the formation of double-membrane structures known as autophagosomes, which engulf α-syn aggregates. The initiation and formation of autophagosomes depend on the activity of several autophagy-related genes (ATGs), including ULK, which is regulated by upstream pathways such as mTOR [95]. During autophagy, class III PI3K complexes, including beclin-1 (BECN1), Vps34, and ATG14, produce phosphatidylinositol 3-phosphate, essential for phagophore expansion and autophagosome maturation [92,96,97]. The autophagosome membrane is further marked by LC3 lipidation (from cytosolic LC3-I to lipidated LC3-II), facilitated by ATG7 and ATG3. LC3-II is integrated into the autophagosomal membrane supports its expansion and subsequent fusion with lysosomes to form autolysosomes, where lysosomal enzymes degrade α-syn aggregates [95,98].

Phagocytosis and autophagy share several components and pathways, notably the involvement of LC3 in the LC3-associated phagocytosis (LAP) and lysosomal fusion for degradation. In LAP, LC3 is recruited to phagosomal membranes, promoting the maturation and fusion of phagosomes with lysosomes, similar to its role in autophagosome formation during autophagy [92,96,97,99,100]. Both processes also rely on the NADPH oxidase 2 complex for ROS generation, which aids in degrading internalized cargo and stimulates further autophagic and phagocytic activity, enhancing α-syn clearance [92,101].

#### 3.4.2. Impairment of α-Syn Clearance Pathways in PD

Notably, in the context of PD, the efficiency of the pathways mentioned in Section 3.4.1 is often compromised due to multiple factors, contributing to disease progression. Studies show that pathological forms of α-syn, particularly those prevalent in PD, significantly impair microglial phagocytosis, disrupting the clearance of extracellular aggregates and exacerbating neuroinflammation [102,103,104].

Rojanathammanee et al. indicated that the A53T α-syn mutation, commonly associated with familial PD, induces a pro-inflammatory microglial phenotype, reducing phagocytic efficiency [102]. Further research demonstrates that microglial responses are diverse and based on α-syn conformations; the wild-type and A53T α-syn can promote microglial phagocytosis, whereas the A30P and E46K variants inhibit this process [103]. Notably, oligomeric α-syn—a form predominant in PD pathology—has been shown to inhibit both basal and stimulated phagocytic activity, preventing efficient α-syn clearance [104]. These findings highlight that the form of α-syn significantly affects microglial function, with pathological variants disrupting normal phagocytosis.

Age is another factor affecting microglial phagocytic capacity. Microglia from older mice exhibited reduced efficiency in engulfing α-syn oligomers compared to those from younger mice while also showing higher levels of TNF-α release [105]. This age-related factor aligns with the increased prevalence of PD in older populations, suggesting that aging further exacerbates phagocytosis dysfunction, contributing to the accumulation of α-syn aggregates.

PD-linked genetic mutations also impair autophagy and lysosomal function. Mutation in genes such as *leucine-rich repeat kinase 2* (*LRRK2*) and *GBA* affect lysosomal enzymes, reducing autophagic capacity and compromising autophagosome–lysosome fusion processes [15,106]. For example, the LRRK2 G2019S mutation, the most common pathogenic form in familial PD, is closely linked to autophagic dysregulation. LRRK2 G2019S knock-in mice and LRRK2 G2019S mutated SH-SY5Y cells show increased LC3-II levels and more autophagic vesicles, indicating autophagic flux inhibition [107,108]. This mutation disrupts autophagosome–lysosome fusion, leading to autophagosome accumulation, insufficient α-syn clearance, and subsequent neuronal damage [108]. Mutations in GBA, which encodes lysosomal enzymes involved in lipid metabolism, are another common PD risk factor [106]. Magalhaes et al. demonstrated that PD patient-derived fibroblasts with GBA1 mutations impaired lysosomal function and disrupted the autophagy–lysosome pathway. This deficiency impedes lysosome reformation from autolysosomes, leading to the accumulation of undigested autophagic material, resulting in cellular stress and increased α-syn aggregation [109]. Together, these impairments collectively result in undigested α-syn aggregates and other damaged organelles, exacerbating neuroinflammation and contributing to dopaminergic neuronal death.

## 4. Therapeutic Approaches and Future Directions

Understanding the pathological mechanisms underlying PD, particularly the interplay between α-syn aggregation and microglial activation, has created new avenues for therapeutic interventions. Several potential therapeutic strategies focus on modulating microglial activation, enhancing clearance mechanisms, and blocking the propagation of α-syn aggregates (Table 1). These strategies are aimed at mitigating neuroinflammation and slowing neurodegeneration, offering the potential for better management of PD symptom management and disease progression control. While these strategies show promise, significant challenges still remain.

### 4.1. Targeting α-Syn Propagation Pathways

Preventing the cell-to-cell propagation of α-syn represents another therapeutic approach, as halting the spread of α-syn aggregates in the brain may slow the progression of PD.

Exosome pathway inhibitors have the therapeutic potential of blocking exosome formation or release, which could limit α-syn spread between cells. GW4869, a neutral sphingomyelinase 2 inhibitor, regulates the production and release of exosomes [18,110,111]. Guo et al. demonstrated that GW4869 treatment reduces exosomes secretion in the microglia exposed to PFF and LPS, limiting exosome-mediated α-syn transfer to neighboring neurons [18]. Tsutsumi et al. also showed that GW4869 reduced dopaminergic neuronal damage and pro-inflammatory cytokine production, indicating that inhibiting exosome secretion can alleviate neuronal injury and inflammation [111].

Additionally, studies have suggested that inhibition of TNTs and TNT-associated molecule formation could prevent the intercellular transfer of α-syn and limit its spread [20,80,81]. While TNT-targeted therapies remain untested in clinical settings, their potential in PD therapy lies in curbing the transmission of pathogenic proteins and reducing neuroinflammatory responses.

### 4.2. Modulating Microglial Activation States

Several therapeutic approaches are currently under investigation and in clinical trials, which aim to modulate the activation state of microglia to reduce inflammation while enhancing α-syn clearance efficiency. These strategies focus on shifting microglia toward a neuroprotective phenotype, thereby promoting clearance of pathological α-syn aggregates while minimizing the pro-inflammatory responses that contribute to neurodegeneration.

The small molecule TAK-242 is a specific TLR4 inhibitor that reduces the production of pro-inflammatory cytokines such as IL-1β and TNF-α by blocking TLR4 signaling. Preclinical models suggest that TAK-242 possesses neuroprotective potential to reduce α-syn-induced microglial activation [112]. Although originally developed for treating inflammatory conditions like sepsis, TAK-242 did not demonstrate sufficient efficacy in phase III clinical trial in sepsis leading to the discontinuation of its clinical development. Currently, TAK-242 is primarily used as a research tool in preclinical studies. Despite promising results in reducing neuroinflammation in preclinical models, no clinical data exist to validate its efficacy in PD. NPT520-34 is a TLR2 antagonist that enhances autophagy to facilitate the clearance of misfolded a-syn. Preclinical studies have demonstrated its effectiveness in reducing α-syn accumulation, decreasing neuroinflammation, and improving motor function in L61 mouse models of PD, highlighting the potential of NPT520-34 as a therapeutic agent to target neuroinflammation and α-syn clearance. NPT520-34 is currently in Phase Ⅰ trials [113]. Another TLR inhibitor, CU-CPT22, targets and inhibits TLR1/2, blocking microglial activation and mitigating neuroinflammation. However, the application of CU-CPT22 remains in the preclinical research stage, where it has demonstrated effectiveness in reducing inflammation and microglial activation triggered by α-syn aggregation [114].

Modulating or inhibiting the NF-κB pathway using compounds such as hypoestoxide and lenalidomide has shown efficacy in reducing microgliosis, protecting dopaminergic neurons, and alleviating motor deficits in PD models [115,116]. Given the role of NF-κB in propagating α-syn pathology, its inhibition offers a promising approach to prevent excessive microglial activation in synucleinopathies. Minocycline, an anti-inflammatory antibiotic, has also shown promise in inhibiting microglial activation and reducing pro-inflammatory cytokines in PD models [117]. However, in an NET-PD study, minocycline did not provide meaningful therapeutic benefits, highlighting the challenges of translating preclinical success into clinical efficacy [118,119].

Lysosomal enhancers aim to enhance cellular mechanisms for clearing α-syn aggregates. Ambroxol enhances lysosomal function and promotes α-syn clearance by increasing glucocerebrosidase (GCase) activity, which is either deficient or dysfunctional in PD patients with GBA mutations [120]. Acting as a molecular chaperone, ambroxol assists in proper GCase folding, improving lysosomal function and the clearance efficiency of α-syn aggregates [121]. Ambroxol is currently in a Phase III clinical trial for PD (NCT05778617), with the ASPro-PD trial (larger, placebo-controlled trial) testing its ability to slow PD progression [122]. Dynasore, a dynamin inhibitor, blocks α-syn aggregate internalization by inhibiting endocytosis in microglia, reducing inflammation in PD models and allowing microglia to retain other protective functions without engulfing α-syn [123]. The autophagy-targeting chimera (AUTOTAC), a chimeric compound composed of a target protein-binding ligand and a p62/SQSTM1-binding ligand, selectively degrades α-syn aggregates by enhancing p62-mediated autophagy pathways [124]. In the PD model, oral administration of ATC161, an AUTOTAC-based drug, reduced α-syn aggregates and their propagation in the brain, alleviating glial inflammatory responses and enhancing motor performances. However, clinical outcomes for PD with this approach have yet to be reported.

**Table 1:** Drugs targeting the α-syn–microglia interaction.

Drugs	Therapeutic Strategy	Efficacy	Status	clinicaltrials.gov ID
Neuroinflammation				
Minocycline	Inhibition of microglial activation	Reduced inflammation in α-syn mouse model [117]; no significant therapeutic effect in early PD [118,119].	Phase II	NCT00063193 (completed)
NPT520-34	Inhibition of microglial activation	Confirmed safety, tolerability, and pharmacokinetic profile in healthy volunteers; planned to evaluate efficacy in PD.	Phase I	NCT03954600 (completed)
TAK-242	TLR4 inhibitor	Shown to suppress microglial activation and reduce neuroinflammation in preclinical models of PD by targeting TLR4 [112].	Preclinical	
CU-CPT22	TLR2 inhibitor	Reduced p-α-syn-induced inflammation and restored autophagic function in an MPTP mouse model [84,114].	Preclinical	
Inzomelid	NLRP3 inflammasome inhibitor	Entered Phase I clinical trials as a potential disease-modifying therapy for PD.	Phase I	NCT04015076 (completed)
α-syn clearance				
Ambroxol	Enhances GCase activity	Increased GCase levels and reduced α-syn accumulation in both patients with and without GBA1 mutations [122].	Phase II	NCT02941822 (completed) NCT06193421 (recruiting) NCT02914366 (Active, not recruiting)
BIA 28-6156	Allosteric activator of GCase	Undergoing evaluation to determine efficacy in patients with GBA1 gene variant.	Phase II	NCT05819359 (Active, not recruiting)
Venglustat (GZ/SAR402671)	Glucosylceramide synthase inhibitor	Reduced α-syn in preclinical models but failed to show efficacy in Phase II trials, leading to discontinuation.	Phase II	NCT02906020 (Terminated)
PR001 (LY3884961)	Enhances GCase activity	Undergoing evaluation to assess the safety, tolerability, immune response, biomarkers, and efficacy in patients.	Phase I/II	NCT04127578 (Recruiting)
ATC161	Enhances autophagy	Reduced brain α-syn aggregates and glial inflammation, improving motor function in a PD model [124].	Preclinical	
α-syn propagation				
GW4869	Neutral sphingomyelinase inhibitor (blocks exosome release)	Reduced α-syn propagation in preclinical studies by inhibiting the release of exosome-contained α-synuclein [111].	Preclinical	-

### 4.3. Personalized and Gene Therapy Approaches

Traditional therapeutic strategies for PD have largely focused on broad-spectrum approaches. However, personalized gene therapy offers a patient-tailored option, particularly for subgroups with specific genetic mutations linked to PD, such as SNCA, LRRK2, or GBA mutations. Although PD exhibits lower genetic diversity across patients than diseases with high genetic heterogeneity, which poses a challenge for personalized treatments, personalized approaches may benefit patients with clear genetic backgrounds [125,126,127,128,129].

CRISPR-Cas9 gene-editing tools, for instance, hold potential for regulating or correcting specific pathogenic genes. For patients with SNCA mutations, CRISPR approaches targeting α-syn expression could help reduce pathogenic α-syn accumulation, potentially mitigating disease progression [130,131]. It could also target other PD-linked genes, such as *LRRK2* and *GBA*, allowing for precise genetic modifications to address specific mutation-related dysfunctions. Another approach involves siRNA-based regulation of α-syn expression. By targeting specific microRNAs, such as miR-155 and miR-let-7a, siRNA could modulate α-syn levels and inflammatory responses simultaneously, offering a less interventional alternative to gene-editing tools while also addressing efficiency and safety challenges associate with more invasive approaches like CRISPR [132,133].

The Adeno-associated-virus (AAV)-based gene delivery system has potential to deliver gene-editing tools or therapeutic genes directly to affected brain regions. AAV vectors have been tested in other neurological diseases, suggesting long-term therapeutic effects that may be beneficial in PD [134,135,136,137]. Recent advancements in enhancing AAV delivery systems are improving both precision and stability for gene therapies. Notably, researchers have focused on enhancing AAV targeting microglia through capsid engineering, promoter modification, and gene silencing; however, challenges remain, particularly in ensuring cell specificity [138,139,140,141,142,143,144]. Therefore, improving AAV precision is crucial, as it may overcome the limitations of traditional AAV methods and enhance the safety of gene therapies in modulating microglial functions during PD pathology.

While precision medicine holds promise, current limitations in identifying reliable biomarkers and the heterogeneity of PD make it challenging. Although researchers are creating processes to identify specific molecular, genetic, and clinical biomarkers, many remain experimental, and their validation for clinical use remains a major hurdle [129]. AI-driven predictive models are emerging as valuable tools for more targeted, personalized approaches by analyzing genetic, molecular, and clinical profiles to stratify patients and optimize therapies, potentially improving outcomes and reducing side effects [25,27,28]. These tools are expected to be essential for refining therapeutic interventions and advancing the goal of personalized care in PD.

### 4.4. Diagnosing and Monitoring PD Progression Using PET Imaging

Positron emission tomography (PET) imaging is used to diagnose and monitor PD progression and assess therapeutic efficacy. Translocator protein (TSPO) PET is used to evaluate microglial activation and facilitates the assessment of disease progression. TSPO, an 18 kDa protein located in the mitochondrial outer membrane in microglia, plays a pivotal role in regulating biological processes related to mitochondrial stress. Additionally, TSPO is a significant biomarker for quantifying inflammatory responses in microglia, with expression markedly elevated upon activation [145]. Increased TSPO levels have been observed in patients with PD [146,147]. PET imaging of TSPO provides a non-invasive method for visualizing and quantitatively assessing microglial activation, offering valuable insights into neuroinflammatory processes linked to PD. Notably, recent PET studies on TSPO indicated that elevated microglial activation may be detectable in the prodromal phases of PD, including in individuals with non-motor symptoms [148]. Xiang et al. developed [^18^F]-F0502B, an α-syn-specific PET tracer for imaging synucleinopathy. F0502B selectively labels α-syn aggregates in mouse and macaque PD models and post-mortem brain tissues of PD patients; however, F0502B has not previously been applied to human subjects [26]. This imaging modality allows clinicians to monitor disease progression and evaluate therapeutic efficacy by comparing microglial activation levels before and following treatment. In summary, the application of PET imaging plays an essential role in understanding pathological alterations in PD and enhancing patient management [149].

## 5. Conclusions

PD is characterized by the complex interplay between pathological α-syn aggregation and microglial activation, both of which contribute to progressive neurodegeneration. Microglia detect α-syn aggregates through various receptors, including TLRs, Fcγs, scavenger receptors, RAGE, and TREM2, and respond to α-syn via multiple pathways. Microglia facilitate the propagation of α-syn through TNTs and exosomes, spreading the α-syn pathology throughout the brain. Subsequently, microglia shift into inflammatory states, activating processes such as NLRP3 inflammasome formation and the UPR. Additionally, microglia play protective roles by clearing α-syn aggregates via phagocytosis and autophagy. However, these clearance processes are often impaired in PD due to factors such as aging, genetic mutations, and pathological α-syn conformations. Thus, maintaining a balance in microglial function is crucial for mitigating α-syn pathology in PD. Therefore, understanding the dual roles of microglia is essential for developing effective therapeutic strategies that modulate their activation states to balance neuroprotection with controlled inflammation.

The therapeutic interventions discussed in this review, including targeting α-syn propagation pathways, enhancing lysosomal function, and modulating microglial activation, show promise in mitigating PD pathology. Recent advances in small-molecule inhibitors, autophagy enhancers, and TLR antagonists highlight the potential of these approaches to directly reduce neuroinflammation and promote α-syn clearance. Moreover, other approaches, such as AAV-based gene therapy, CRISPR–Cas9 gene editing, and personalized therapies tailored to individual genetic profiles offer avenues for more targeted, long-lasting treatments. However, while preclinical studies present encouraging results, many of these strategies still face challenges in clinical application, particularly in achieving efficient targeting of microglia, optimizing delivery systems, and minimizing adverse effects. Addressing these challenges will be key to realizing the therapeutic potential of these approaches. Additionally, PET imaging could aid in diagnosing PD and monitoring disease progression and treatment efficacy. In summary, while recent advances present viable possibilities, significant challenges in biomarker validation, delivery optimization, safety, specificity, and clinical translation must be addressed to ensure these therapies become safe, effective, and accessible for PD patients.

This review summarizes the complex interaction between microglia and α-syn in PD, highlighting the therapeutic potential of approaches targeting these mechanisms. Further research is needed to refine these strategies, particularly to better understand how to balance the neuroinflammatory and neuroprotective functions of microglia. Developing future therapies that modulate the interaction between microglia and α-syn could offer more effective ways to slow disease progression and improve the quality of life for patients with PD.
